# Unexpectedly large thrombi in the left atrium detected with intracardiac echocardiography in a case after left atrial appendage ligation

**DOI:** 10.1016/j.hrcr.2025.01.017

**Published:** 2025-02-05

**Authors:** Masataka Narita, Hitoshi Mori, Yoshifumi Ikeda, Naomichi Tanaka, Wataru Sasaki, Ritsushi Kato

**Affiliations:** Department of Cardiology, Saitama Medical University, International Medical Center, Saitama, Japan

**Keywords:** Thrombi in the left atrium, Catheter ablation, Intracardiac echocardiography, Mitral valve replacement, Left atrial appendage ligation, Preoperative examination

**Key Teaching Points**

• The number of atrial fibrillation ablation procedures is increasing, and pre-ablation thrombus evaluation is crucial. However, detailed investigations on thrombus evaluation after surgical left atrial appendage closure have not been thoroughly conducted.
• We report a rare case of a large thrombus on the left atrial surface during an ablation procedure, as observed via intracardiac echocardiography, despite the maintenance of sinus rhythm after surgical left atrial appendage ligation.
• This highlights the necessity of preoperative examination for thrombus, even after mitral valve replacement surgery combined with surgical left atrial appendage exclusion.

## Introduction

The incidence of atrial fibrillation (AF) is gradually increasing because of the aging society.^1^ Various methods for preventing cerebral embolisms, including anticoagulants, percutaneous left atrial appendage (LAA) closure, surgical LAA ligation, and surgical LAA resection, have been widely performed.^2^ Surgical LAA exclusion is an excellent treatment that effectively reduces the incidence of strokes. Therefore, it is increasingly performed during mitral valve surgery and surgical pulmonary vein isolation. The “2020 ACC/AHA guideline for the management of patients with valvular heart disease” states that postoperative anticoagulants are reasonable for at least 3 months after a surgical LAA exclusion.^3^ After the mandatory 3-month period, anticoagulation therapy is not necessarily required. The “2017 HRS/EHRA/ECAS/APHRS/SOLAECE expert consensus statement on catheter and surgical ablation of atrial fibrillation” states that, in cases in which AF persists before performing percutaneous catheter ablation, a detailed examination for LAA thrombi using transesophageal echocardiography is required.^4^ A pre-ablation thrombus evaluation is not always necessary when sinus rhythm is maintained. In actual clinical practice, in many cases thrombus evaluation is not performed. We report a rare case following an LAA ligation, in which large thrombi were detected in the LA during the ablation procedure by intracardiac echocardiography (ICE).

## Case report

An 80-year-old woman was referred to our hospital for the treatment of symptomatic uncommon atrial flutter (AFL). The CHADS₂ (congestive heart failure, hypertension, age of or greater than 75 years, or diabetes mellitus, and history of stroke) score was 2 points, with the patient meeting the criteria for age and heart failure. She did not have any familial history of pro-thrombotic conditions. Four years earlier, she had undergone an aortic valve replacement (Insiris RESILIA 21 mm; Edwards Lifesciences, Irvine, CA), mitral valve replacement (Mosaic 25 mm; Medtronic, Minneapolis, MN), LAA ligation (Atri Clip 35 mm; AtriCure, Inc., Mason, OH), and Maze procedure for aortic stenosis, mitral regurgitation, and paroxysmal AF, respectively. She discontinued warfarin 3 months after the surgery. During that time, her international normalized ratio levels ranged between 1.6 and 2.6.

Immediately after the initial diagnosis of uncommon AFL, an appropriately low dose of dabigatran therapy (110 mg twice daily) was initiated, considering the specific pharmacokinetics and smaller body size of Japanese patients. Transthoracic echocardiography showed that left ventricular systolic function was preserved at over 50%, and there was no left ventricular enlargement. The left atrial (LA) diameter was 45 mm, and the LA volume was 89.5 mL. There were no findings of prosthetic valve insufficiency in the aortic or mitral valve. No obvious thrombi in the heart were observed within the field of view. Two months later, after her initial visit to our department, she returned to the outpatient department, reporting palpitations and dizziness, with a home-recorded heart rate of 140 beats/min. Despite a preserved left ventricular systolic function, there was a risk of worsening heart failure because of tachycardia. She was urgently admitted to the hospital, and direct current electrical cardioversion was performed on the same day without conducting an LAA thrombus evaluation, because she was on anticoagulant therapy and had undergone an LAA ligation. This successfully restored sinus rhythm.

**Figure 1:**
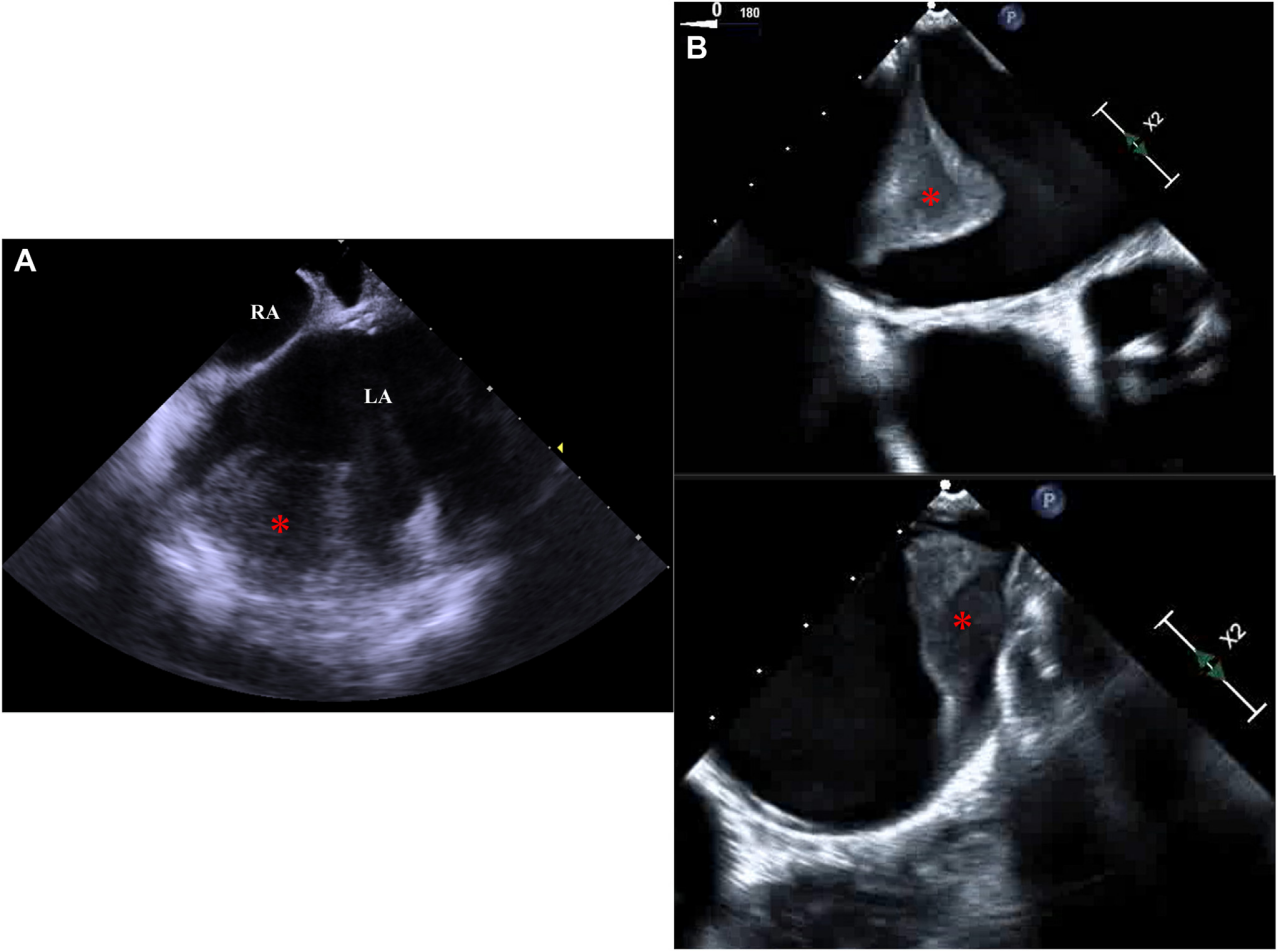
**A:** Intracardiac echocardiography revealed large thrombi (*asterix*) in the LA before the atrial septal puncture. **B:** Transesophageal echocardiography was performed to evaluate the LA thrombi; the 2 thrombi were 40.5 mm × 23.9 mm and 26.7 mm × 15.8 mm, respectively. RA = right atrium; LA = left atrium.

**Figure 2:**
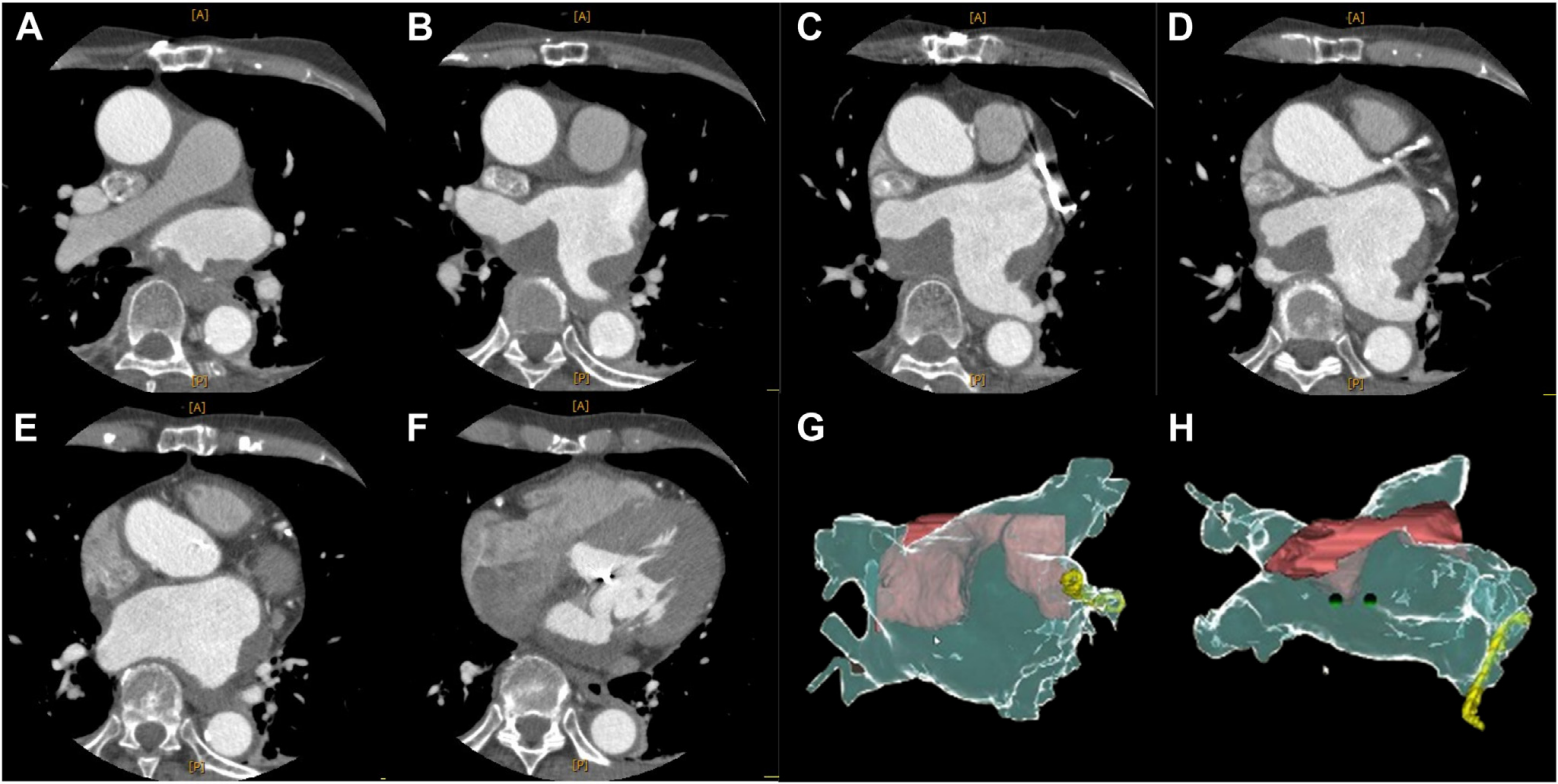
**A–F:** The CT images show that the thrombus is extensively attached from the posterior wall to the lateral wall of the LA. **G, H:** The 3D image of the thrombus: the red color represents the thrombus, extending from the posterior to the lateral wall. CT = computed tomography; LA = left atrium.

**Figure 3:**
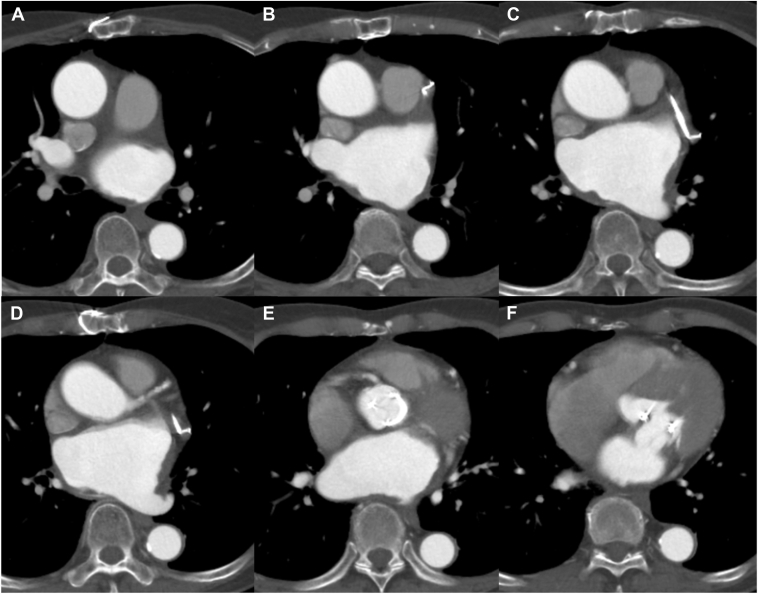
The computed tomography images obtained 3 months later revealed partial thrombus remnants on the lateral and posterior walls, but a reduction in thrombus size was observed.

One month after this emergent cardioversion, she was readmitted for planned catheter ablation of AF and AFL. Her electrocardiogram showed sinus rhythm during this readmission, and ablation was performed without a thrombus evaluation. The blood test indicated elevated serum N-terminal pro-brain natriuretic peptide (1869 pg/mL) and D-dimer (6.2 ng/mL). After securing the vascular access, an ICE catheter (AcuNav™, Biosense Webster, Inc., Irvine, CA) was positioned in the right atrium to assist in the transseptal puncture, which showed a highly echogenic mass on the surface of the LA, suggestive of a thrombus (Figure 1A). The procedure was canceled, and a detailed evaluation of this mass was performed. Transesophageal echocardiography revealed the presence of 2 large thrombi in the LA, measuring 40.5 mm × 23.9 mm and 26.7 mm × 15.8 mm, respectively (Figure 1B). Cardiac computed tomography (CT) angiography showed those thrombi had adhered to the LA’s posterior wall, lateral wall, bottom, and roof (Figure 2, Supplemental video 1). Anticoagulation therapy was switched from dabigatran to warfarin with an international normalized ratio level between 2.0 and 3.0. She was discharged home in a stable condition. We checked the background thrombosis conditions. The patient had no history of malignant tumors, and contrast-enhanced CT did not reveal any large masses. Blood tests showed that total homocysteine, protein C, and protein S levels were within the normal range, and antinuclear antibodies were negative. Lupus anticoagulant and anti-cardiolipin antibodies were also within the normal range. After 3 months of anticoagulant therapy, a CT scan revealed a reduction in the thrombus (Figure 3, Supplemental video 2). Anticoagulant therapy with warfarin was continued, and the patient has been progressing without any embolic events.

## Discussion

In most AF cases, thrombi are formed in the LAA.^5^ To prevent thrombus formation, surgical LAA resection and ligation have been widely performed during surgical procedures. In the current case, however, thrombi were extensively attached to the body of the LA, even after the LAA ligation. Two possible causes were considered for the thrombus formation after the LAA surgery.

The first possibility was the presence of a residual LAA or pouch. Thrombus formation can occur when a large pouch of 1 cm or more remains after an LAA exclusion.^6^ However, no obvious LAA or pouch was observed on imaging in the current case, making it an unlikely cause.

The second possibility involved thrombus formation in the LA after the mitral valve replacement surgery. Jindong Chen et al^7^ reported that LA thrombi were identified in 45 cases (1.4%) of 3117 cases after mitral valve replacement surgery. This report noted that the distribution of left thrombi was as follows: roof (n = 10), posterior (n = 9), left atrial appendage (n = 7), lateral (n = 6), anterior (n = 2), septal (n = 1), and multiple sites (n = 10). The thrombi group had 40 cases (88.9%) of atrial fibrillation or atrial flutter, whereas the control group had 70.6%, which was significantly higher.^7^ All thrombi were fixed at the base and were immobile. Independent predictors of an LA thrombus occurrence include a left atrial size >55 mm, increased mitral valve pressure gradient (>6 mm Hg), and left ventricular ejection fraction (<50%).^7^ Although none of these factors existed in this case, characteristics of thrombus formation after the mitral valve replacement surgery were evident. Additionally, endocardial lesions resulting from the Maze procedure can contribute to thrombus formation.^8^

A thrombus evaluation is not necessarily performed before ablation in cases in which the LAA has been resected or ligated. However, as seen in this case, there are instances where thrombus formation occurs in the body of the LA. For patients with risk factors for LA thrombi, it is necessary to consider a preoperative thrombus evaluation. Furthermore, even in patients without risk factors for LA thrombi, our case suggested the possibility of thrombus formation within the LA.

Tsurugi et al^9^ reported that a thrombus evaluation using intraoperative ICE is effective during ablation procedures, even in cases following an LAA resection. A thrombus evaluation of the LA body using ICE before the septal puncture may be useful in preventing thromboembolic events.^9^ The use of anticoagulants after surgical LAA closure should be carefully considered. The 2020 ESC Guidelines for the Diagnosis and Management of Atrial Fibrillation underscore the necessity for adequately powered trials to evaluate the optimal antithrombotic therapy after surgical LAA occlusion or exclusion.^10^ As such, a standardized approach has yet to be established. However, our case highlights the possibility that continuing anticoagulation therapy should be considered, particularly in patients with a high CHADS2 score.

## Conclusion

Unexpected large thrombi were found in the LA body using an ICE image in a case with AF after a surgical LAA exclusion. Preoperative examination for thrombi is necessary, even after mitral valve replacement combined with left atrial appendage exclusion. ICE may be useful for evaluating the thrombus during the procedure.

## Disclosures

None
