# Supplementary material for: Unexpectedly large thrombi in the left atrium detected with intracardiac echocardiography in a case after left atrial appendage ligation
Source: HeartRhythm Case Rep. 2025 Feb 5;11(5):395–9. doi: 10.1016/j.hrcr.2025.01.017 (PMC12242957; doi:10.1016/j.hrcr.2025.01.017)
Supplement: Supplemental Video Legends [file mmc1.docx]

**Supplemental Video 1.**

A contrast-enhanced CT scan shows the left atrium from the head to the tail. There was a large area of thrombi attached to the wall.

**Supplemental Video 2.**

The CT images obtained three months later revealed partial thrombus remnants on the lateral and posterior walls, but a reduction in thrombus size was observed.
